# Patient-specific Deformation Modelling via Elastography: Application to Image-guided Prostate Interventions

**DOI:** 10.1038/srep27386

**Published:** 2016-06-07

**Authors:** Yi Wang, Dong Ni, Jing Qin, Ming Xu, Xiaoyan Xie, Pheng-Ann Heng

**Affiliations:** 1 Department of Computer Science and Engineering, The Chinese University of Hong Kong, Shatin, New Territories, Hong Kong, China; 2 Guangdong Key Laboratory for Biomedical Measurements and Ultrasound Imaging, School of Biomedical Engineering, Shenzhen University, Shenzhen 518060, China; 3 Shenzhen Institutes of Advanced Technology, Chinese Academy of Science, Shenzhen, China; 4 Centre for Smart Health, School of Nursing, The Hong Kong Polytechnic University, Hong Kong, China; 5 Department of Medical Ultrasonics, the First Affiliated Hospital, Institute of Diagnostic and Interventional Ultrasound, Sun Yat-Sen University, Guangzhou, China

## Abstract

Image-guided prostate interventions often require the registration of preoperative magnetic resonance (MR) images to real-time transrectal ultrasound (TRUS) images to provide high-quality guidance. One of the main challenges for registering MR images to TRUS images is how to estimate the TRUS-probe-induced prostate deformation that occurs during TRUS imaging. The combined statistical and biomechanical modeling approach shows promise for the adequate estimation of prostate deformation. However, the right setting of the biomechanical parameters is very crucial for realistic deformation modeling. We propose a patient-specific deformation model equipped with personalized biomechanical parameters obtained from shear wave elastography to reliably predict the prostate deformation during image-guided interventions. Using data acquired from a prostate phantom and twelve patients with suspected prostate cancer, we compared the prostate deformation model with and without patient-specific biomechanical parameters in terms of deformation estimation accuracy. The results show that the patient-specific deformation model possesses favorable model ability, and outperforms the model without patient-specific biomechanical parameters. The employment of the patient-specific biomechanical parameters obtained from elastography for deformation modeling shows promise for providing more precise deformation estimation in applications that use computer-assisted image-guided intervention systems.

Prostate cancer is the most common noncutaneous cancer and the second leading cause of cancer death in men^1^. Currently, the routine clinical modality for imaging the prostate, especially for image-guided prostate biopsy and treatments, is transrectal ultrasound (TRUS) because it is safe, portable, and inexpensive. However, some challenges still face surgeons when performing TRUS-guided prostate interventions. One of them is how to locate the targets accurately, given the poorly distinguishing capability of tumors using TRUS imaging. In practice, we can solve this problem either by accurately predicting the prostate deformation and precisely practicing the intervention in a simulation before the interventions or by fusing preoperative magnetic resonance (MR) images with the TRUS images to increase the accuracy of the interventions. Both of these computer-assisted solutions need an accurate model to estimate the prostate deformation so that the surgeons can easily track the targets and perform the operation. However, developing such a deformation model is difficult. Deformations of the prostate are inevitable and various during TRUS imaging because of the insertion of TRUS probe (see Fig. 1), such diverse deformations that occur in TRUS images are difficult to compensate when performing the MR-TRUS registration. On the other hand, each patient’s prostate tissue has specific biomechanical properties, especially when there are pathological changes within the prostate. Furthermore, different regions of the prostate gland may have different biomechanical properties^2^, making the deformation of this inhomogeneous gland difficult to estimate.

In the last decade, prostate deformation modeling has been investigated as a solution for image-guided prostate interventions. Two main methodological categories have been studied: biomechanical modeling and statistical modeling. Because it is easy to implement, the mass-spring model (MSM) initially came to dominate the biomechanical models^3^,^4^. However, MSMs are usually not consistent with the governing equations of biomechanical systems and thus could not produce reliable results for deformation estimation. The other stream of biomechanical models uses finite element (FE) methods. Bharatha *et al.*^5^ employed a linear elastic materials model to estimate the deformation between pre- and intraoperative prostate images. However, linear models cannot adequately model large deformations of the prostate^6^. When non-linear models are applied, the time performance of FE methods is usually not satisfactory for intraoperative guidance^7^. In addition, acquiring patient-specific biomechanical parameters to ensure the accuracy of FE models is difficult.

On the other hand, statistical modeling methods have been proposed for predicting the deformation by analyzing a set of training data and generating a statistical model, which can be applied in real-time applications. Dam *et al.*^8^ trained the prostate shape model from real patient data. However, collecting training data from a large number of patients is very difficult. Later, Hu *et al.*^9^ proposed an FE-based statistical motion model (SMM) to predict prostate deformation. Unfortunately, the biomechanical parameters that were used to generate the deformation instances were randomly sampled within a specific range. This limitation may reduce the robustness of this method, especially because pathological changes may cause the biomechanical parameters to be outside the predetermined range.

Nevertheless, combining a statistical model with a biomechanical model with patient-specific parameters shows promise for achieving an adequate model of prostate deformation. Because collecting a sufficient number of representatives of the population as training data is essential for building an effective statistical model but collecting a large number of patient data is often difficult in clinical practice, biomechanical modeling shows promise as a way to generate sufficient data for statistical analysis. On the other hand, to achieve a physically appropriate biomechanical model, determining the biomechanical parameters of prostate tissue is important^10^. However, the tissue properties of prostate vary from one person to another, and even appear quite distinctive in the different prostate zones of the same subject. Traditional methods either employ a specific value obtained from certain reports of biomechanical experiments or else apply randomly sampled values within a wide range to build the biomechanical model. Unfortunately, neither scheme is sufficient to build an accurate model for a patient-specific system. The former is obviously not patient-specific, and the latter is not sufficiently robust, especially when there are pathological changes in the prostate. In recent years, shear wave elastography (SWE)^2^ has emerged as an important and widely available imaging modality for lesion detection through tissue elastic variations, and shown to be a valuable complementary tool to the conventional TRUS and MR for prostate diagnosis. Unlike conventional elastography methods, which measure relative stiffness, SWE computes the quantitative shear modulus via the shear wave propagation velocity through the tissues. Furthermore, compared with traditional elastography techniques, SWE is able to measure the quantitative shear modulus with high intra- and inter-observer reproducibility, and thus is less operator-dependent. Therefore, we employed SWE to measure the *in vivo* biomechanical parameters of the prostate and assigned them to an FE model to form a patient-specific deformation model.

The main contribution of this research is to investigate the impact of employing patient-specific biomechanical parameters obtained from SWE data for prostate deformation modeling, by comparing the performance of deformation model constructed with and without patient-specific biomechanical parameters. We further implemented a non-linear elastic material model to describe the non-linear stress-strain behavior of the prostate when undergoing TRUS-probe-induced deformations. Details are described in the method section. The experimental results show that the patient-specific deformation model outperforms the model without patient-specific biomechanical parameters in terms of deformation estimation accuracy.

## Results

### Impact of biomechanical parameter setting on deformation modeling

**Figure 2:**
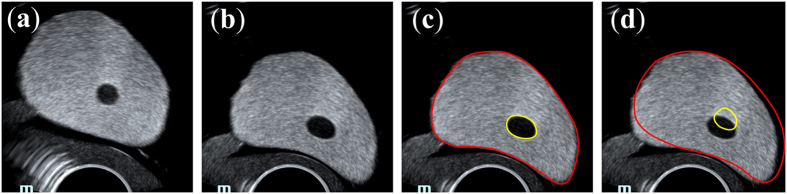
The deformation modeling on prostate phantom 053-AEF. (**a**) TRUS prostate image without probe-induced deformation, (**b**) prostate image with the in-plane deformation caused by probe insertion, (**c**) the accurate deformation modeling of the prostate (red) and inner lesion (yellow) by adopting patient-specific biomechanical parameters, (**d**) the mismatched deformation modeling by using improper biomechanical parameters.

**Figure 3:**
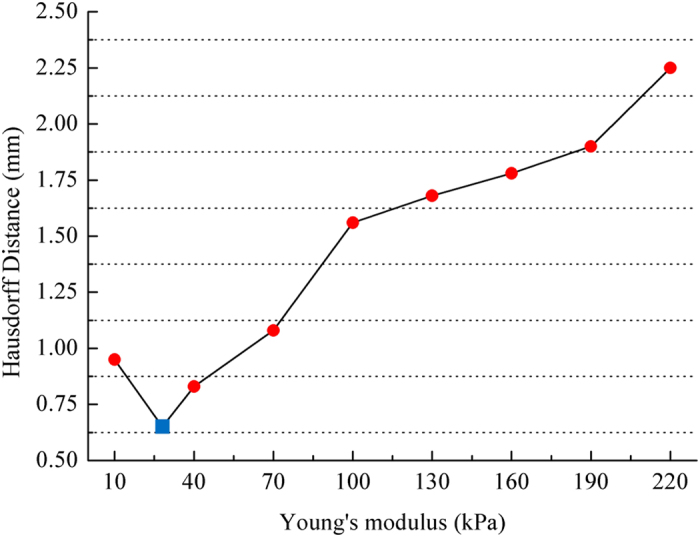
Comparison of the modeled deformations via different parameter settings and the realistic deformation. The blue square denotes the result with the SWE measurements.

The 2D prostate phantom images with and without probe induced deformation are shown in Fig. 2. The Young’s modulus of the phantom prostate gland is 28 kPa obtained with SWE. The hypoechoic region inside the prostate is a synthetic lesion with Young’s modulus of 17 kPa. By adopting these biomechanical values, together with the calculated probe insertion information, the deformation of prostate was modeled and illustrated in Fig. 2(c). The prostate gland, as well as the inner lesion region, were deformed more realistically and similarly toward the realistic deformation, whereas the modeling deficiency with improper tissue parameter (100 kPa) can be found in Fig. 2(d). On the other hand, Fig. 3 shows the Hausdorff distance values between various modeled deformations via different Young’s moduli and the realistic deformation. As shown in Fig. 3, the most accurate deformation modeling can be achieved with the parameters measured from the SWE. Furthermore, it can be observed that when the Young’s modulus was progressively deviated away from the patient-specific value, the modeled deformation also gradually derailed from the realistic deformation. It can be found in the Figs 2 and 3 that the use of the phantom-specific parameter measured from SWE attains the best modeling result that is closest to the real case.

### Impact of patient-specific deformation model on MR-TRUS registration

To illustrate the effect of biomechanical parameter setting on the deformation estimation, we systematically perturbed the biomechanical parameters of inner and outer prostate with positive and negative offsets from the SWE measures for the model-guided MR-TRUS registration on all the patient data. For the perturbing of outer prostate, the parameter of inner prostate was fixed as SWE the measurement, whereas the outer prostate parameter was similarly unchanged while perturbing the parameter of inner prostate.

**Figure 4:**
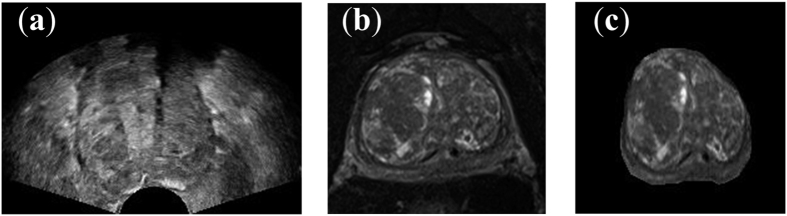
Example slices: (**a**) the TRUS image, (**b**) the MR image, (**c**) the registered MR image using our deformation model.

Figure 4 visualizes one target TRUS slice, MR slice and the corresponding registered MR slice obtained using the patient-specific deformation model. The detailed relations between the parameter settings on inner and outer prostate and resulted registration performances of each patient are listed in Tables 1 and 2. Specifically, the mean and standard deviation of TRE values are reported. Table 3 further summarizes the registration performances with respect to the changes of inner and outer tissue parameters, and demonstrates the statistical significance (*p*-values of two-tailed student tests) between the registration results of using changed tissue parameter and unchanged SWE measurement.

It can be observed from Tables 1 and 2 that the employment of the SWE measures of the inner and outer prostate can averagely yield satisfactory deformation estimation accuracy. It can also be observed from Table 3 that for the setting of biomechanical parameters, the performance differences of target registration will be statistically significant if the defined parameter is averagely deviated beyond 15 kPa from the SWE measurement.

## Discussion

We proposed a deformation model for estimating patient-specific prostate deformation during image-guided interventions. We employed shear wave elastography to obtain the patient-specific biomechanical parameters. Coupled with a non-linear elastic material model, these biomechanical parameters are beneficial for generating a realistic deformation model for practical applications. The experimental results demonstrated the superior performance of our proposed deformation model over the traditional model without patient-specific biomechanical parameters in terms of deformation estimation accuracy.

**Figure 5:**
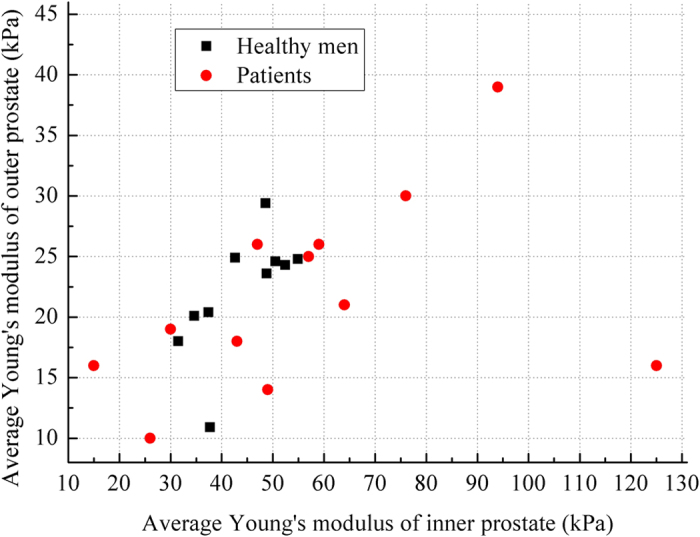
Variation in the Young’s moduli of the inner and outer prostate glands from different men. The black squares denote healthy men, and the red dots denote patients with suspected prostate cancer.

Precise measurements of tissue properties have been shown to be crucial for a realistic biomechanical modeling^7^,^10^. To demonstrate the necessity of adopting patient-specific parameters for the accurate deformation modeling, we conducted twofold experiments. First, a prostate phantom-based validation experiment was conducted. The different deformation results in Fig. 2 demonstrate the importance of setting precise biomechanical parameters for deformation modeling. Furthermore, it can be observed from Fig. 3 that the most accurate deformation modeling can be attained with the patient-specific parameters, whereas deficient deformation results happened when modeling with improper biomechanical parameters. Second, we conducted model-guided MR-TRUS registrations to compare the deformation model with and without patient-specific biomechanical parameters in terms of deformation estimation accuracy. The comparison results in Tables 1 and 2 demonstrate that the patient-specific deformation model with SWE measurement is able to provide the most accurate deformation estimation for MR-TRUS registration. Table 3 further demonstrates the performance differences of the MR-TRUS registration can be statistically significant if the biomechanical setting is deviated beyond 15 kPa from the patient-specific value. Figure 5 displays the Young’s modulus for the inner and outer prostate glands of 10 healthy men and 12 patients and shows that the biomechanical parameters vary greatly between the two different prostate regions, as well as between different subjects. This further indicates the importance and necessity of employing patient-specific biomechanical parameters in deformation estimations.

Although the deformation model we developed in this study was primarily designed for the purpose of estimating prostate deformation, it is a general modeling method that makes use of SWE data and has the potential for being applied in other clinical applications for the following reasons. First, shear wave elastography is widely utilized in clinical diagnosis and has become a valuable method for imaging the prostate, liver, thyroid, breast, etc., complementing conventional ultrasound and MR^11^. Second, combined biomechanical and statistical modeling methods have already been used for estimating the deformation of breast^12^ and liver^13^. However, these methods often used the same biomechanical properties for different patients, a practice which may lead to an inaccurate deformation estimation due to large individual differences. Therefore, since *in vivo* personalized biomechanical parameters can be acquired by readily accessible SWE, our patient-specific deformation modeling method should be easily adapted for these clinical applications to improve their accuracy of deformation estimation.

## Methods

### Data acquisition

Experiments were carried out on the datasets obtained from a prostate phantom and twelve patients with suspected prostate cancer at the First Affiliated Hospital of Sun Yat-Sen University. The study protocol was reviewed and approved by the Ethics Committee of Sun Yat-Sen University and informed consent was obtained from all patients. The methods were carried out in accordance with the approved guidelines. A set of MR, TRUS, and SWE data were acquired from each patient. The MR images were applied to construct the geometric model of the prostate; the SWE data were employed to obtain the patient-specific biomechanical parameters; the TRUS images that showed the deformed prostate were used to evaluate the deformation estimation performance of the proposed deformation model through the MR-TRUS registration. The T2-weighted MR images were acquired using a 3.0 Tesla Siemens TrioTim MR-scanner (Erlangen, Germany) with a 32 channels body coil. The MR voxel size was 0.625 × 0.625 × 3.6 mm^3^ in x-, y- and z-direction. The 3D TRUS images were obtained by a Mindray DC-8 ultrasound system (Shenzhen, China) with an integrated 3D TRUS probe. The TRUS voxel size was 0.5 × 0.5 × 0.5 mm^3^ in x-, y- and z-direction. The shear wave elastography images were acquired using a Supersonic Aixplorer (Aix-en-Provence, France) ultrasound system. The Supersonic Aixplorer provides the Q-Box tool which can calculate the average Young’s modulus within an operator selected region. The average Young’s moduli of inner and outer prostate were obtained separately by sampling several Q-Boxes within the corresponding glands. To ensure the accuracy, the Q-Boxes were selected by experienced doctors.

### Patient-specific biomechanical modeling

**Figure 6:**
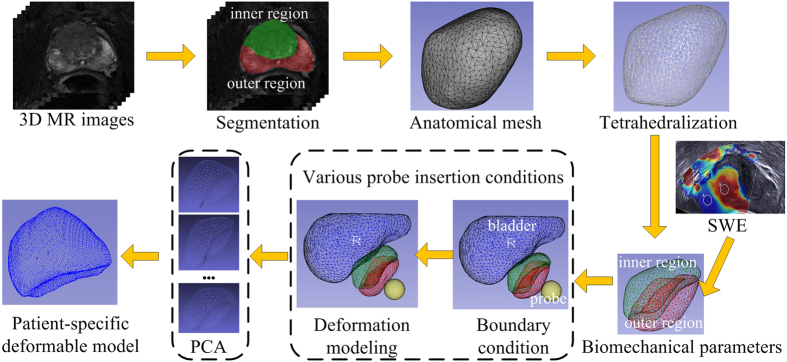
Workflow of patient-specific deformation modeling.

Our modeling framework consisted of two steps (see Fig. 6). We first constructed a patient-specific biomechanical model based on anatomical meshes derived from MR images and biomechanical parameters acquired from ultrasound elastography. Then, we used principal component analysis (PCA) to generate a statistical deformation model from a set of patient-specific biomechanical models with randomly sampled boundary conditions.

When undergoing image-guided interventions, the prostate often deforms, primarily due to the insertion of the TRUS probe. Previous mass-spring and finite element methods modeled using linear elastic models have not been able to simulate such large deformations. In our implementation, we assumed that the involved tissues are elastic/hyperelastic isochoric materials and employed a neo-Hookean model to formulate the biomechanical behaviors of the prostate. Many studies have applied this model to predict the non-linear stress-strain behaviors of materials undergoing large deformations^14^. We briefly introduce this model and its key parameters here; readers can refer to^14^ for more details. In the neo-Hookean model, the strain energy density *W*_*s*_ is formulated as:


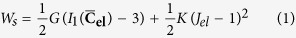


where 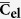 is the isochoric-elastic right Cauchy-Green deformation tensor, 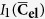 is the first invariant of 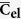 and is equal to the trace of 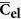, *J*_*el*_ is the elastic volume ratio, *G* is the shear modulus and *K* is the bulk modulus. The 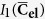 and *J*_*el*_ can be calculated from the elastic deformation tensor **F**_**el**_:


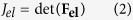



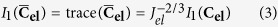


where **C**_**el**_ = **F**_**el**_^*T*^**F**_**el**_, is the elastic right Cauchy-Green deformation tensor and 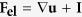 (**u** is the displacement vector and **I** is the unit matrix). On the other hand, with the strain energy density *W*_*s*_, the second Piola-Kirchhoff stress can be calculated as:


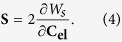


Finally, the displacement **u** can be figured out from the **S** via *W*_*s*_. Combining equations (1, 2, 3, 4), it is clearly that the specific deformation of the prostate can be calculated given the patient-specific biomechanical parameters (*G* and *K*) and the boundary conditions (prostate geometry and external forces). Details of the implementation follow.

Assigning an accurate shear modulus *G* and bulk modulus *K* to the solver is essential. These two moduli are material-dependent and can be calculated by Young’s modulus *E* and Poisson’s ratio *ν*:


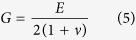


and


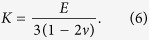


**Figure 7:**
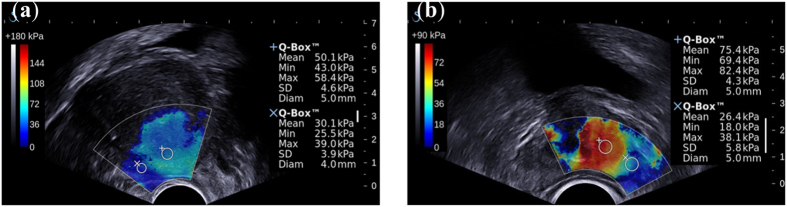
Prostate shear wave elastography images from two patient. The +Q-Box and ×Q-Box located in the inner and outer glands, respectively, yielded considerably different stiffness measures. The stiffness also varied greatly from patient to patient.

For the Poisson’s ratio *ν*, we assume that the prostate is nearly incompressible and simply set it to be a constant 0.495^15^. However, acquiring an accurate Young’s modulus *E* for a specific patient can be challenging, especially because different regions of the prostate have different biomechanical characteristics. In addition, these properties vary from patient to patient^2^. In this research we employed quantitative shear wave elastography (SWE)^16^ to obtain a patient-specific Young’s modulus of the prostate. Figure 7 shows the prostate shear wave elastography images from two patients. The stiffness of the inner and outer glands is obviously different, but within either the inner or outer gland, the change in stiffness is quite small. In addition, the stiffness of the prostate varied greatly from patient to patient. These observations are consistent with a previous study on the biomechanical properties of the prostate^2^. We obtained the Young’s modulus for the inner and outer glands of each patient from the acquired SWE data by averaging the Young’s modulus values within the inner gland and within the outer gland. Finally, the shear modulus *G* and bulk modulus *K* were calculated and assigned to the neo-Hookean model.

To obtain the information for the geometric model, we segmented the prostate and bladder gland using data from MR images via an interactive segmentation software: SmartPaint^17^. In clinical practice, the prostate is usually divided into two parts: the inner gland and the outer gland, which have obviously different biomechanical properties. For this reason, in order to model the biomechanical behaviors of the prostate more accurately, an experienced physician further segmented and refined the outer and inner gland regions. The refined segmentation results were converted into triangulated surface meshes using an adaptive skeleton climbing method^18^, which can overcome the gap-filling problem in traditional marching cubes algorithms. Then the noise on the surface meshes was removed by employing a recently developed coarse-to-fine normal filtering scheme^19^. Thus, patient-specific geometric models with accurate anatomic features and relationships were generated.

The boundary conditions define the external forces and restrictions exerted on the prostate during TRUS-guided interventions. The insertion of the TRUS probe is the main cause of prostate deformation. To realistically model the interactions between the TRUS probe and the prostate, the front end of the TRUS probe was reconstructed based on its physical shape. Then the prostate and 3D TRUS probe models were set as mechanical contact pairs in our implementation. Other boundary conditions, such as the patient’s position and the pelvic bone surface, have little influence on the deformation of the prostate^20^. Therefore, we did not consider these boundary conditions in our implementation.

We employed COMSOL, an FE analysis software, to calculate the deformation of the prostate. We input the governing equations and the material-dependent parameters to the non-linear FE solver embedded in COMSOL. In our implementation environment, each deformation modeling took about 15 seconds to calculate the mesh displacement.

### Combined statistical and biomechanical modeling

Although the anatomic geometry and biomechanical parameters are patient-specific for deformation modeling, the conditions of the TRUS probe insertion are very difficult to measure *in vivo*. To this end, we further employed the statistical modeling method to analyze the deformations under different probe insertion situations. To collect data that was representative of the population for statistical modeling, we conducted *Q* (*Q* = 100 is an empirical value in our implementation) biomechanical modelings for each patient using the same anatomic model and biomechanical parameters, but different settings for the probe insertion conditions. Through such personalized biomechanical modeling, each modeled result was able to efficiently represent the patient-specific prostate deformation induced by a particular insertion of the TRUS probe. Afterwards, the patient-specific deformation model for each patient was calculated by statistically analyzing the *Q* modeled deformation instances using PCA method.

### Validation of the impact of patient-specific biomechanical parameter setting on deformation modeling

Two experiments were conducted to validate the impact of patient-specific biomechanical parameter setting on deformation modeling.

#### Impact of biomechanical parameter setting on deformation modeling

To analyze the effect of the biomechanical parameter setting on the accuracy of deformation modeling, a validation experiment was first carried on one prostate phantom Model 053-AEF (CIRS, Norfolk, USA). Two specific TRUS image sets were acquired for the quantitative analysis on the efficacy of using patient-specific biomechanical parameters. First, one TRUS prostate image set, denoted as P1, without probe-induced deformation was obtained and manually segmented as the basis for the parameterized deformation modeling. The other TRUS image set (P2) was acquired with the in-plane deformation caused by the probe insertion. The position of the TRUS probe was recorded by the electromagnetic tracking sensor attached to the probe, thus the probe movement information can be accurately calculated. Our objective is to illustrate that whether the deformation modeling result on P1 by adopting patient-specific biomechanical parameters is similar to the realistic prostate deformation on P2. To quantitatively demonstrate the influence of the biomechanical parameter setting for deformation modeling, eight uniformly sampled elasticity values from a specific range ([9, 230] kPa) of tissue parameter as recommended in Hu’s work^9^ were used to generate the respective deformation results. Meanwhile, the deformation modeling with the biomechanical parameter measured with SWE was also performed. We further compared the realistic deformation (P2) and the modeling results with the Hausdorff distance that can quantitatively illustrate the differences between the modeled and realistic phantom boundaries.

#### Application to MR-TRUS registration for prostate interventions

We employed the patient-specific deformation model to perform model-guided MR-TRUS registration^21^ on twelve patients, which is of high interest in image-guided intervention systems, to evaluate its ability to estimate the deformation. Given the manually segmented TRUS and MR prostate surface point sets, we integrated the patient-specific deformation model into a robust point matching (RPM) framework^22^ to register MR surface with TRUS surface in order to realize the volumetric prostate deformation. The registration algorithm proceeds by (1) establishing correspondence between MR and TRUS surface point sets using RPM, and (2) by estimating the deformation required to register the corresponding surface points using proposed deformation model. Processes (1) and (2) are embedded within an annealing scheme to dually update for registering MR surface with TRUS surface. The dual process is ended until certain Temperature is reached. Finally, based on the registered surfaces, the MR images can be warped to the TRUS images.

The target registration error (TRE)^23^, defined as the Euclidean distance between corresponding, manually-identified intrinsic landmarks in MR and TRUS images, was measured to evaluate the accuracy of the model-based registration. All the landmarks used for the TRE calculation were manually extracted by a urological physician with extensive experience in interpreting MR and TRUS prostate images. The locations of centers that corresponded to small nodules, cysts, and calcifications inside the prostate were selected as landmarks in both the MR and TRUS images. For each patient, 4–6 pairs of corresponding landmarks were manually extracted. And totally 61 pairs of landmarks were extracted from twelve patients for the TRE calculation.

## Additional Information

**How to cite this article**: Wang, Y. *et al.* Patient-specific Deformation Modelling via Elastography: Application to Image-guided Prostate Interventions. *Sci. Rep.* **6**, 27386; doi: 10.1038/srep27386 (2016).

## Figures and Tables

**Figure 1:**
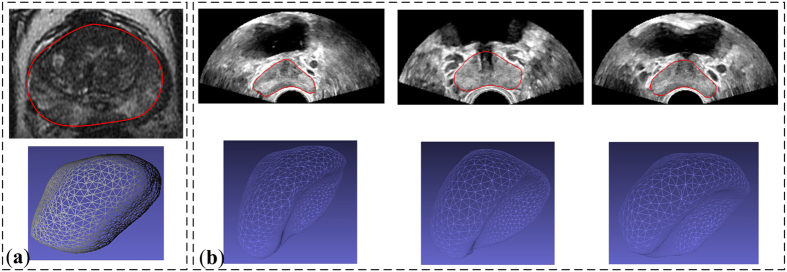
MR and TRUS prostate images. (**a**) MR image and 3D prostate surface model without probe-induced deformation, (**b**) TRUS images and 3D surface models with various probe-induced deformations.

**Table 1:** Detailed relations between the biomechanical parameter settings of inner prostate and the registration performances on each patient data.

Patient Case 1: Young’s modulus of outer prostate = 26 kPa											
Young’s modulus of inner prostate (kPa)	32	37	42	***47***	52	57	62	97	147	197	247
TRE: mean (SD)	2.76 (0.45)	2.40 (0.47)	2.32 (0.46)	***2.22*** (***0.54***)	2.22 (0.55)	2.39 (0.43)	2.47 (0.45)	2.62 (0.52)	2.72 (0.58)	2.88 (0.68)	3.01 (0.71)
Patient Case 2: Young’s modulus of outer prostate = 19 kPa											
Young’s modulus of inner prostate (kPa)	15	20	25	***30***	35	40	45	80	130	180	230
TRE: mean (SD)	3.05 (0.45)	2.56 (0.41)	2.18 (0.40)	***2.07*** (***0.43***)	2.16 (0.42)	2.23 (0.42)	2.31 (0.42)	2.58 (0.37)	2.73 (0.38)	2.76 (0.40)	2.93 (0.47)
Patient Case 3: Young’s modulus of outer prostate = 25 kPa											
Young’s modulus of inner prostate (kPa)	7	42	47	52	***57***	62	67	72	107	157	207
TRE: mean (SD)	2.73 (0.46)	2.04 (0.19)	1.90 (0.19)	1.80 (0.14)	***1.55*** (***0.36***)	1.77 (0.21)	1.87 (0.24)	1.94 (0.30)	2.09 (0.24)	2.16 (0.25)	2.22 (0.27)
Patient Case 4: Young’s modulus of outer prostate = 16 kPa											
Young’s modulus of inner prostate (kPa)	25	75	110	115	120	***125***	130	135	140	175	225
TRE: mean (SD)	2.78 (0.32)	2.55 (0.27)	2.30 (0.28)	2.22 (0.29)	2.17 (0.30)	***2.13*** (***0.31***)	2.18 (0.30)	2.28 (0.28)	2.37 (0.27)	2.61 (0.28)	2.71 (0.26)
Patient Case 5: Young’s modulus of outer prostate = 26 kPa											
Young’s modulus of inner prostate (kPa)	9	44	49	54	***59***	64	69	74	109	159	209
TRE: mean (SD)	2.75 (0.23)	2.30 (0.32)	2.19 (0.26)	2.11 (0.31)	***2.07*** (***0.29***)	2.15 (0.22)	2.18 (0.23)	2.29 (0.22)	2.42 (0.27)	2.49 (0.26)	2.55 (0.31)
Patient Case 6: Young’s modulus of outer prostate = 18 kPa											
Young’s modulus of inner prostate (kPa)	28	33	38	***43***	48	53	58	93	143	193	243
TRE: mean (SD)	2.44 (0.37)	2.30 (0.38)	2.12 (0.31)	***2.10*** (***0.33***)	2.21 (0.27)	2.19 (0.32)	2.25 (0.31)	2.30 (0.38)	2.32 (0.40)	2.39 (0.41)	2.44 (0.35)
Patient Case 7: Young’s modulus of outer prostate = 39 kPa											
Young’s modulus of inner prostate (kPa)	44	79	84	89	***94***	99	104	109	144	194	244
TRE: mean (SD)	2.33 (0.35)	2.05 (0.31)	2.01 (0.36)	1.93 (0.33)	***1.88*** (***0.33***)	1.92 (0.30)	1.97 (0.29)	2.03 (0.32)	2.30 (0.31)	2.33 (0.28)	2.45 (0.28)
Patient Case 8: Young’s modulus of outer prostate = 10 kPa											
Young’s modulus of inner prostate (kPa)	11	16	21	***26***	31	36	41	76	126	176	226
TRE: mean (SD)	2.42 (0.23)	2.35 (0.22)	2.31 (0.24)	***2.22*** (***0.22***)	2.19 (0.24)	2.29 (0.22)	2.35 (0.20)	2.41 (0.22)	2.49 (0.22)	2.53 (0.18)	2.57 (0.20)
Patient Case 9: Young’s modulus of outer prostate = 16 kPa											
Young’s modulus of inner prostate (kPa)		5	10	***15***	20	25	30	65	115	165	215
TRE: mean (SD)		2.22 (0.44)	2.19 (0.42)	***2.19*** (***0.44***)	2.25 (0.45)	2.28 (0.46)	2.28 (0.45)	2.35 (0.45)	2.39 (0.43)	2.45 (0.45)	2.52 (0.50)
Patient Case 10: Young’s modulus of outer prostate = 14 kPa											
Young’s modulus of inner prostate (kPa)	34	39	44	***49***	54	59	64	99	149	199	249
TRE: mean (SD)	2.12 (0.28)	2.11 (0.30)	2.05 (0.26)	***2.02*** (***0.28***)	2.05 (0.28)	2.11 (0.28)	2.08 (0.32)	2.16 (0.28)	2.20 (0.33)	2.28 (0.32)	2.28 (0.31)
Patient Case 11: Young’s modulus of outer prostate = 21 kPa											
Young’s modulus of inner prostate (kPa)	14	49	54	59	***64***	69	74	79	114	164	214
TRE: mean (SD)	2.62 (0.49)	2.32 (0.47)	2.31 (0.43)	2.29 (0.46)	***2.22*** (***0.48***)	2.23 (0.45)	2.27 (0.44)	2.31 (0.47)	2.47 (0.46)	2.45 (0.47)	2.53 (0.44)
Patient Case 12: Young’s modulus of outer prostate = 30 kPa											
Young’s modulus of inner prostate (kPa)	26	61	66	71	***76***	81	86	91	126	176	226
TRE: mean (SD)	2.41 (0.32)	2.15 (0.35)	2.10 (0.35)	2.07 (0.37)	***2.05*** (***0.36***)	2.06 (0.32)	2.08 (0.32)	2.13 (0.33)	2.24 (0.36)	2.29 (0.35)	2.41 (0.33)

Values in bold italic represent the Young’s modulus measured by SWE.

**Table 2:** Detailed relations between the biomechanical parameter settings of outer prostate and the registration performances on each patient data.

Patient Case 1: Young’s modulus of inner prostate = 47 kPa											
Young’s modulus of outer prostate (kPa)	11	16	21	***26***	31	36	41	76	126	176	226
TRE: mean (SD)	2.48 (0.50)	2.34 (0.47)	2.20 (0.43)	***2.22*** (***0.54***)	2.26 (0.45)	2.37 (0.49)	2.49 (0.51)	2.92 (0.65)	2.97 (0.69)	3.08 (0.78)	3.08 (0.87)
Patient Case 2: Young’s modulus of inner prostate = 30 kPa											
Young’s modulus of outer prostate (kPa)	4	9	14	***19***	24	29	34	69	119	169	219
TRE: mean (SD)	2.78 (0.56)	2.49 (0.37)	2.28 (0.38)	***2.07*** (***0.43***)	2.00 (0.36)	2.37 (0.41)	2.58 (0.40)	2.97 (0.33)	3.04 (0.35)	3.26 (0.35)	3.36 (0.33)
Patient Case 3: Young’s modulus of inner prostate = 57 kPa											
Young’s modulus of outer prostate (kPa)	10	15	20	***25***	30	35	40	75	125	175	225
TRE: mean (SD)	1.85 (0.26)	1.78 (0.23)	1.68 (0.25)	***1.55*** (***0.36***)	1.73 (0.25)	1.86 (0.26)	1.89 (0.35)	2.26 (0.32)	2.27 (0.44)	2.40 (0.37)	2.38 (0.60)
Patient Case 4: Young’s modulus of inner prostate = 125 kPa											
Young’s modulus of outer prostate (kPa)	1	6	11	***16***	21	26	31	66	116	166	216
TRE: mean (SD)	2.33 (0.33)	2.21 (0.28)	2.16 (0.28)	***2.13*** (***0.31***)	2.18 (0.40)	2.22 (0.34)	2.34 (0.33)	2.49 (0.26)	2.78 (0.32)	2.85 (0.33)	2.92 (0.31)
Patient Case 5: Young’s modulus of inner prostate = 59 kPa											
Young’s modulus of outer prostate (kPa)	11	16	21	***26***	31	36	41	76	126	176	226
TRE: mean (SD)	2.25 (0.27)	2.15 (0.27)	2.09 (0.29)	***2.07*** (***0.29***)	2.09 (0.26)	2.14 (0.30)	2.20 (0.27)	2.58 (0.33)	2.63 (0.36)	2.69 (0.31)	2.77 (0.42)
Patient Case 6: Young’s modulus of inner prostate = 43 kPa											
Young’s modulus of outer prostate (kPa)	3	8	13	***18***	23	28	33	68	118	168	218
TRE: mean (SD)	2.43 (0.33)	2.26 (0.33)	2.18 (0.29)	***2.10*** (***0.33***)	2.14 (0.33)	2.43 (0.27)	2.59 (0.26)	2.64 (0.28)	2.68 (0.33)	2.75 (0.37)	2.81 (0.40)
Patient Case 7: Young’s modulus of inner prostate = 94 kPa											
Young’s modulus of outer prostate (kPa)	24	29	34	***39***	44	49	54	89	139	189	239
TRE: mean (SD)	2.01 (0.35)	1.98 (0.34)	1.90 (0.27)	***1.88*** (***0.33***)	1.93 (0.23)	1.95 (0.31)	2.01 (0.29)	2.28 (0.27)	2.32 (0.26)	2.37 (0.25)	2.42 (0.25)
Patient Case 8: Young’s modulus of inner prostate = 26 kPa											
Young’s modulus of outer prostate (kPa)			5	***10***	15	20	25	60	110	160	210
TRE: mean (SD)			2.24 (0.18)	***2.22*** (***0.22***)	2.27 (0.25)	2.42 (0.23)	2.58 (0.17)	2.63 (0.19)	2.67 (0.18)	2.64 (0.23)	2.76 (0.22)
Patient Case 9: Young’s modulus of inner prostate = 15 kPa											
Young’s modulus of outer prostate (kPa)	1	6	11	***16***	21	26	31	66	116	166	216
TRE: mean (SD)	2.31 (0.40)	2.27 (0.44)	2.27 (0.45)	***2.19*** (***0.44***)	2.20 (0.47)	2.26 (0.47)	2.27 (0.45)	2.31 (0.47)	2.38 (0.43)	2.45 (0.39)	2.46 (0.39)
Patient Case 10: Young’s modulus of inner prostate = 49 kPa											
Young’s modulus of outer prostate (kPa)		4	9	***14***	19	24	29	64	114	164	214
TRE: mean (SD)		2.09 (0.27)	2.06 (0.28)	***2.02*** (***0.28***)	2.03 (0.28)	2.07 (0.25)	2.09 (0.26)	2.35 (0.20)	2.40 (0.22)	2.40 (0.17)	2.52 (0.09)
Patient Case 11: Young’s modulus of inner prostate = 64 kPa											
Young’s modulus of outer prostate (kPa)	6	11	16	***21***	26	31	36	71	121	171	221
TRE: mean (SD)	2.31 (0.44)	2.25 (0.43)	2.21 (0.40)	***2.22*** (***0.48***)	2.27 (0.44)	2.28 (0.44)	2.31 (0.46)	2.79 (0.37)	2.87 (0.40)	2.92 (0.41)	3.00 (0.41)
Patient Case 12: Young’s modulus of inner prostate = 76 kPa											
Young’s modulus of outer prostate (kPa)	15	20	25	***30***	35	40	45	80	130	180	230
TRE: mean (SD)	2.35 (0.34)	2.23 (0.35)	2.13 (0.34)	***2.05*** (***0.36***)	2.07 (0.33)	2.11 (0.37)	2.17 (0.32)	2.32 (0.33)	2.56 (0.34)	2.63 (0.33)	2.75 (0.35)

Values in bold italic represent the Young’s modulus measured by SWE.

**Table 3:** The registration performances with respect to the changes of inner and outer prostate parameters.

	Inner prostate		Outer prostate	
Young’s moduli offsets (kPa)	TRE: mean (SD)	*t*-test: *p*-value	TRE: mean (SD)	*t*-test: *p*-value
−100	2.78 (0.32)	0.0018	–	–
−50	2.54 (0.36)	1.97E-07	–	–
−15	2.37 (0.45)	0.0001	2.32 (0.44)	0.0013
−10	2.23 (0.38)	0.0196	2.20 (0.37)	0.0599
−5	2.13 (0.35)	0.3216	2.12 (0.34)	0.3854
0	2.07 (0.38)	1.0	2.07 (0.38)	1.0
5	2.12 (0.35)	0.4462	2.10 (0.35)	0.5962
10	2.18 (0.34)	0.0778	2.21 (0.37)	0.0392
15	2.24 (0.35)	0.0100	2.29 (0.39)	0.0015
50	2.39 (0.37)	4.79E-06	2.55 (0.41)	4.32E-10
100	2.46 (0.39)	1.33E-07	2.65 (0.43)	1.5E-12
150	2.51 (0.41)	2.07E-08	2.72 (0.46)	3.67E-14
200	2.66 (0.51)	1.03E-6	2.79 (0.49)	2.88E-15

The p-values of student tests demonstrate the statistical significance between the registration results of using changed parameter and unchanged SWE measurement.
